# Capsaicin Inhibits Biofilm and Its Related Functions in *Helicobacter pylori*

**DOI:** 10.3390/microorganisms14061293

**Published:** 2026-06-08

**Authors:** Khalid I. AlHussaini, Razique Anwer

**Affiliations:** 1 Department of Internal Medicine, College of Medicine, Imam Mohammad Ibn Saud Islamic University (IMSIU), Riyadh 4233-13317, Saudi Arabia; 2 Department of Pathology, College of Medicine, Imam Mohammad Ibn Saud Islamic University (IMSIU), Riyadh 4233-13317, Saudi Arabia

**Keywords:** capsaicin, *Helicobacter pylori*, biofilm, molecular simulation

## Abstract

Background: *Helicobacter pylori* is a globally prevalent gastric pathogen associated with chronic gastritis, peptic ulcer disease, and gastric adenocarcinoma. Its persistence within the gastric niche is strongly linked to biofilm formation, contributing to immune evasion and antibiotic therapy resistance. Methodology: In the present study, we investigated the antibiofilm potential of capsaicin, a natural phytochemical derived from Capsicum species, against *H. pylori* using experimental and computational approaches. Results: Capsaicin treatment significantly reduced biofilm biomass (up to 75.66 ± 4.00%), metabolic activity (up to 61.23 ± 6.88%), and cell surface hydrophobicity in a dose-dependent manner. Microscopic analyses revealed disrupted biofilm architecture and diminished extracellular polymeric substance at higher concentrations. Molecular docking analysis revealed that capsaicin interacts with target *H. pylori* proteins (GTP cyclohydrolase II, α-carbonic anhydrase, and urease) through stable hydrogen bonds and hydrophobic contacts. Molecular dynamics simulations further supported the stability of these complexes and demonstrated reduced structural fluctuations upon ligand binding. Free energy landscape analysis suggested ligand-induced conformational alterations in α-carbonic anhydrase, indicating possible structural effects associated with capsaicin interaction. Conclusions: Overall, the findings provide insight into the antibiofilm activity of capsaicin against *H. pylori* and highlight its potential as a natural adjunct strategy for combating biofilm-associated persistence and antimicrobial resistance.

## 1. Introduction

*Helicobacter pylori* is a spiral-shaped Gram-negative bacterium colonizing human gastric mucosa and is recognized as one of the prevalent chronic infections. It is estimated that nearly half of the global population harbours *H. pylori*, with developing countries reporting higher prevalence [1]. Persistent colonization is linked to numerous gastrointestinal diseases, such as peptic ulcer disease, chronic gastritis, gastric adenocarcinoma, and mucosa-associated lymphoid tissue (MALT) lymphoma [2]. Despite the availability of antibiotic-based therapies, treatment failure is common, largely due to increasing resistance to first-line agents such as clarithromycin (macrolide) and metronidazole (nitroimidazole), as well as second-line drugs including levofloxacin (fluoroquinolone) and tetracycline [3]. Patient’s negligence and the bacterium’s ability to adapt to the harsh gastric environment further contribute to severity of such *H. pylori* infections [4].

One of the key survival strategies of *H. pylori* is its ability to make biofilms on the gastric mucosa. Biofilms are structured microbial communities encased in an extracellular polymeric substance (EPS) matrix that provides defence against environmental stress, host immune responses, and antimicrobial agents [5]. Within biofilms, bacterial cells exhibit altered physiology, reduced metabolic activity, and enhanced genetic exchange, all of which contribute to persistence and chronic infection [6]. In the case of *H. pylori*, biofilm formation facilitates long-term colonization of gastric epithelium and is directly linked to therapeutic failure. Biofilm-embedded cells are less susceptible to antibiotics compared to planktonic cells, frequently requiring more drug doses for eradication [7]. Moreover, biofilms serve as reservoirs for resistant genes, accelerating the emergence of multidrug resistance. Previous studies have shown that *H. pylori* biofilm-forming cells exhibit higher expression of efflux pump genes such as *hp1165* and *hefA*, leading to reduced susceptibility to antibiotics including amoxicillin, metronidazole, and erythromycin [8]. Similarly, clinical isolates with biofilm phenotypes display increased resistance profiles against clarithromycin, metronidazole, and levofloxacin compared to planktonic cells [9,10]. These findings highlight the role of biofilms as genetic reservoirs that facilitate persistence and multidrug resistance. Recent studies have also highlighted that *H. pylori* biofilms not only impair antibiotic penetration but also modulate quorum sensing pathways, further enhancing virulence and adaptability [11]. Thus, targeting biofilm formation represents a promising approach to overcome drug resistance and mitigate the effects of *H. pylori* infections.

Capsaicin, a pungent compound in chilli peppers (*Capsicum* spp.), is a member of the capsaicinoid class of vanilloid alkaloids. The compound is widely studied for its diverse biological activities. Beyond its well-known role in modulating pain perception through TRPV1 activation [12], capsaicin exhibits antioxidant, anti-inflammatory, anticancer, and antimicrobial properties [13]. Several reports have found its bacteriostatic ability against Gram-negative and Gram-positive bacteria [13], and that it disrupts membrane integrity and interferes with quorum sensing pathways [14]. Its relatively low toxicity and dietary origin make capsaicin an attractive candidate for therapeutic applications. Capsaicin has been reported to exert antimicrobial and antibiofilm effects in several bacterial species [15]; its activity against *H. pylori* biofilms remains poorly understood. Although direct epidemiological evidence linking dietary capsaicin consumption to reduced infection risk is limited, capsaicin has been extensively studied for its antimicrobial and antibiofilm properties, providing a rationale for its evaluation against *H. pylori* biofilms. Moreover, no study has yet evaluated the effect of capsaicin established biofilms and nor have its molecular interactions with critical *H. pylori* enzymes such as urease, GTP cyclohydrolase II, and α-carbonic anhydrase been explored. These enzymes as the known targets with urease required for acid survival, α-CA for pH regulation, and GCHII for metabolic support. This will give detailed ideas about the interaction of capsaicin with these proteins. Therefore, we hypothesized that capsaicin could serve as a natural inhibitor of biofilm formation, thereby reducing bacterial survival and pathogenicity. By integrating in vitro assays with molecular simulations, this study provides insights into capsaicin’s antibiofilm potential and evaluates its promise as a natural therapeutic candidate against *H. pylori* infection.

## 2. Materials and Methods

### 2.1. Bacterial Strain and Its Growth Condition

*Helicobacter pylori* ATCC 43504 was used to check the effect of capsaicin in this study. The strain was obtained from ATCC and maintained under microaerophilic conditions. The culture was grown on Brain Heart Infusion (BHI) agar supplemented with 7% sheep blood serum. For liquid culture experiments, *H. pylori* was grown in BHI containing 10% fetal bovine serum (FBS) at 37 °C under microaerophilic conditions (5% O_2_, 85% N_2_, and 10% CO_2_,) using a tri-gas incubator (Thermo Fisher Scientific, Waltham, MA, USA). Fresh cultures were prepared by sub-culturing from agar plates into broth medium and incubating for 48 h to reach the mid-logarithmic growth phase. Cell density was monitored spectrophotometrically at 600 nm (OD_600_) to standardize inoculum size for subsequent assays. Capsaicin was obtained from Merck (Cat no. 12084), and its stock solution of 20 mM was prepared in DMSO. The final concentration of DMSO was ≤0.5% *v*/*v* in all treatments.

### 2.2. MIC Determination

The MIC of capsaicin against *H. pylori* ATCC 43504 was determined using broth microdilution method, following standard method with minor modifications [16]. The inoculum of *H. pylori* of approximately 1 × 10^6^ CFU/mL was placed in wells of sterile 96-well polystyrene plates containing various capsaicin dilutions. Capsaicin stock was diluted in BHI broth supplemented with 10% FBS to obtain final concentrations ranging from 25 to 1600 µM. Wells containing inoculated broth without capsaicin served as growth controls. The solvent control DMSO (0.5% *v*/*v*) was also taken. The plate was incubated at 37 °C under microaerophilic conditions for 48 h. The MIC was defined as the lowest concentration of capsaicin that showed no visible bacterial growth. Further, the absence of growth or metabolically active cells was confirmed by the addition of 10 µL TTC (2,3,5-triphenyltetrazolium chloride) dye. TTC is reduced by active bacterial cells to insoluble red formazan, thereby serving as a measure of respiratory activity. The presence of metabolically active cells turns dye to a dark pink or red colour.

### 2.3. Growth Kinetics

The effect of capsaicin on growth kinetics of *H. pylori* ATCC 43504 was evaluated using broth culture assays. Capsaicin from stock DMSO was diluted in BHI broth containing 10% FBS and capsaicin (100–400 µM). The *H. pylori* inoculum of nearly 1 × 10^6^ CFU/mL was placed into 96-well microtiter plates containing capsaicin-supplemented broth. The wells without capsaicin served as growth controls. The effect of 0.5% DMSO was also tested on the bacterial growth kinetics. Plates were incubated at 37 °C under microaerophilic conditions for 48 h. Bacterial growth was monitored spectrophotometrically by measuring optical density at 600 nm (OD_600_) at regular intervals. The growth curves were plotted to compare the control with capsaicin-treated cultures.

### 2.4. Inhibition of Biofilm Formation

The inhibition of *H. pylori* ATCC 43504 biofilm by capsaicin was examined using crystal violet staining [17]. Briefly, standardized bacterial inoculum was dispensed into sterile 96-well flat-bottom microtiter plates containing BHI broth supplemented with 10% FBS and varying doses of capsaicin (10–100 µM). The wells containing *H. pylori* ATCC 43504 without capsaicin served as positive controls. The plates were incubated in microaerophilic conditions at 37 °C for 48 h to allow biofilm formation. Following incubation, the unbound planktonic cells were gently aspirated, and wells were gently washed with sterile PBS to remove unattached bacteria. The wells were air-dried, and adherent biofilm was stained with 0.1% (*w*/*v*) crystal violet solution for 20 min. The excess stain was washed with PBS, and bound dye was dissolved in 95% ethanol. The biofilm was quantified by recording the optical density at 570 nm using a microplate reader. Percent biofilm inhibition was calculated relative to the control.

### 2.5. Microscopy of Biofilm Inhibition

To visualize the effect of capsaicin on *H. pylori* ATCC 43504 biofilms, microscopic analysis was performed using light microscopy (Olympus BX60F5, Tokyo, Japan) and confocal microscopy (Zeiss LSM780, Oberkochen, Germany). The sterile glass coverslips were placed in 12-well tissue culture plates containing bacterial suspension in BHI broth supplemented with 10% FBS. Capsaicin (100 µM) was added to the wells, while untreated wells served as controls. The plate was incubated at 37 °C under microaerophilic conditions for 48 h to allow biofilm formation on coverslip surfaces.

For light microscopy, the coverslips were gently washed with PBS to remove planktonic cells. The biofilms on the coverslip were air-dried for 15 min and stained with 0.1% crystal violet for 20 min. The excess dye was rinsed with PBS, and then the coverslip was air-dried. The stained coverslips were examined under light microscope at 40× magnification to examine the biofilms. For confocal microscopy, the biofilms on coverslips were stained with acridine orange for 15 min in the dark. The stained coverslips were visualized under a confocal microscope with excitation at 488 nm.

### 2.6. Eradication of Biofilm

The ability of capsaicin to eradicate pre-formed *H. pylori* ATCC 43504 biofilms was evaluated using crystal violet staining. Briefly, bacterial inoculum was placed into sterile 96-well flat-bottom microtiter plates containing BHI broth and 10% FBS. The bacteria was incubated at 37 °C under microaerophilic conditions for 48 h to allow for the development of biofilm. After completion of incubation, the unattached cells were removed by washing with sterile PBS. Fresh medium containing varying concentrations of capsaicin (10–100 µM) was then added to the wells, followed by incubation for an another 24 h or 48 h. After treatment, wells were washed with PBS to remove planktonic cells. The biofilm was air-dried and stained with 0.1% crystal violet solution for 20 min. The excess stain was washed with PBS, and the biofilm-attached dye was dissolved in 95% ethanol. The optical density was then recorded at 570 nm using a microplate reader. Percentage eradication of biofilm was calculated relative to untreated control.

### 2.7. Measurement of Metabolic Activity in Biofilms

The metabolic activity of *H. pylori* ATCC 43504 biofilms in response to capsaicin treatment was assessed using the XTT reduction assay [18]. Mature biofilms were established in sterile 96-well flat-bottom microtiter plates by incubating bacterial inoculum in BHI broth containing 10% FBS under microaerophilic conditions at 37 °C for 48 h. After biofilm formation, planktonic cells were discarded by aspiration, and wells were washed with PBS. Capsaicin (10–100 µM) was added to wells, followed by incubation for 24 h under identical conditions. Following treatment, the medium was aspirated, and each well was added with XTT solution (200 µL) containing menadione. The XTT (1 mg/mL) was freshly prepared in phosphate buffer, and menadione stock solution of 1 mM was made separately in acetone. Immediately before the experiment, the working reagent was made by mixing 12.5 mL of the XTT solution with 1 mL of the menadione stock. The plates were incubated at 37 °C in the dark for 3 h, allowing for a reduction in XTT by metabolically active cells. The colorimetric change (orange formazan product) was quantified by taking absorbance at 490 nm. Metabolic activity is expressed as percent inhibition relative to control.

### 2.8. Hydrophobicity Index

The cell surface hydrophobicity of *H. pylori* ATCC 43504 biofilms after capsaicin treatment was evaluated using the microbial adhesion to hydrocarbons assay [19]. Briefly, bacteria were cultured without and with various concentrations of capsaicin (10–100 µM). The bacteria were also cultured with 0.5% DMSO that served as solvent control while untreated culture was used for control. Following 24 h growth, the bacterial cells were harvested, washed twice with PBS (pH 7.0), and then resuspended for an optical density of 0.5 at 600 nm. Aliquots of 3 mL bacterial suspension were added to 1 mL toluene in sterile glass tubes. The mixtures were vortexed properly and then incubated for 15 min to allow for phase separation. The aqueous phase was taken, and its absorbance was measured at 600 nm. The hydrophobicity index was calculated using the formula:

$$Hydrophobicity\;index\;\left(\%\right)=\frac{{OD}_{initial}-{OD}_{aqueous}}{{OD}_{initial}}\times 100$$

where *OD*_initial_ is the absorbance of the bacterial suspension before mixing with toluene, and *OD*_aqueous_ is the absorbance of the aqueous phase after phase separation.

### 2.9. Molecular Docking

Molecular docking was performed to investigate interactions of capsaicin with three key target *H. pylori* proteins, GTP cyclohydrolase II (PDB ID: 4RL4) [20], α-carbonic anhydrase (PDB ID: 4XFW) [21], and urease. First, the molecular docking procedure was validated by extracting the known inhibitor 2-{[1-(3,5-dimethylphenyl)-1H-imidazol-2-yl]sulfanyl}-N-hydroxyacetamide (SHA) from the crystal structure and then redocking. The docked ligand was found at the same site (RMSD = 1.446 Å) as it was present in the crystal structure, thereby validating the docking procedure. The same docking parameters were used for subsequent docking of capsaicin with target proteins. The protein’s 3D structures were taken from the Protein Data Bank. Prior to docking, protein structures were prepared using AutoDock Tools-1.5.6. Preparation involved removing the water, adding polar hydrogens, and assigning Kollman charges. The structure of capsaicin was obtained from PubChem (CID: 1548943) in SDF format and saved as PDBQT. Energy minimization of the ligand was carried out in an MMFF94 force field to optimize geometry and reduce steric clashes. Rigid docking was conducted using AutoDock Vina-1.1.2 [22]. For each protein, the grid box was made to cover the entire receptor molecules. The grid dimensions are listed in Table 1 with a spacing of 1.0 Å. The exhaustiveness was set to 100 to allow for adequate sampling of conformations. The docking output was analyzed based on binding affinity and the best-scoring poses were visualized using PyMOL-3.1 and Discovery Studio 2025.

### 2.10. Molecular Dynamics Simulations

The MD simulations were performed to investigate the structural stability and dynamic behaviour of *H. pylori* target proteins (GTP cyclohydrolase II, α-carbonic anhydrase, and urease) in both apo forms and in a complex with capsaicin. The complex of SHA with urease was also included for simulation that served as a positive control. The least-energy docking poses were used as starting structures for the protein–ligand complexes. All simulations were carried out using GROMACS 2018.1 with the amber99sb-ILDN force field [23,24]. The topology files for capsaicin or SHA were generated using the Antechamber package within AmberTools [25], and atomic charges were assigned using the AM1-BCC charge model. Each system (apo protein and protein–capsaicin complex) was solvated in a cuboidal box with a minimum 10 Å buffer using the TIP3P water model. Neutralization was achieved by adding the counter-ions, and physiological ionic strength was mimicked by including 150 mM NaCl. Energy minimization was performed using the steepest descent algorithm with a cut-off radius of 12 Å for non-bonded interactions to eliminate steric clashes and unfavourable contacts. Equilibration was conducted in two stages. The NVT ensemble was done for 1000 ps at 310 K using the V-rescale thermostat [26]. The NPT ensemble was also done for 1000 ps at 1 bar using the Parrinello–Rahman barostat [27]. Production MD simulations were run for 100 ns under periodic boundary conditions using the LINCS algorithm to constrain bond lengths. Three independent replicates were performed for each system, and the average values with SD are reported. Prior to analysis, periodic boundary condition (PBC) corrections were applied to all trajectories. Additionally, MM-PBSA calculations were performed using the g_mmpbsa package to estimate binding free energies and decompose contributions from van der Waals, electrostatic, polar solvation, and non-polar solvation terms [28].

### 2.11. Statistical Analysis

All in vitro assays were performed in four independent replicates, and data are presented as mean ± standard deviations (SDs). Statistical significance was assessed using one-way analysis of variance (ANOVA) followed by Dunnett’s post hoc test for multiple comparisons, comparing each treatment group to the control group. Statistical significance was defined as *p* < 0.05. The following symbols indicate significance levels: ns (not significant) at *p* ≥ 0.05; * at *p* < 0.05; ** at *p* < 0.0, and 1; *** at *p* < 0.001. Three independent runs of molecular dynamics simulations were performed and data is presented as average with standard deviations.

## 3. Results and Discussion

### 3.1. MIC Determination and Growth Kinetics

Using the broth microdilution method, the MIC of capsaicin against *H. pylori* ATCC 43504 was determined to be 200 µM. At this concentration, visible bacterial growth was completely inhibited, confirming the effective antimicrobial potential of capsaicin.

**Figure 1:**
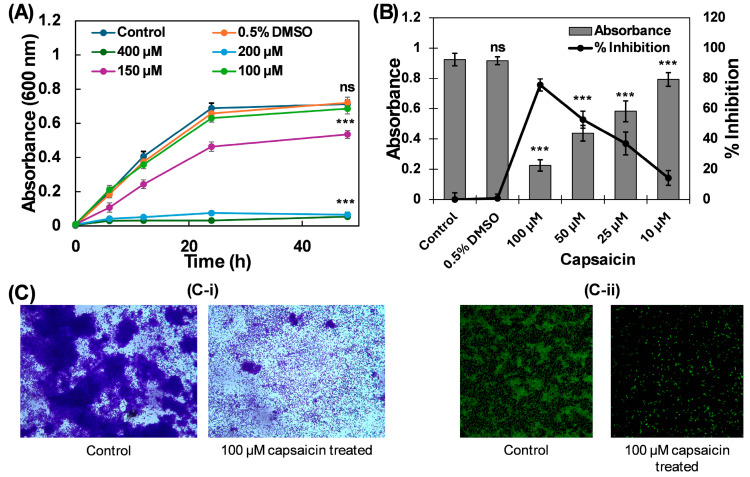
(**A**) Growth kinetics of *H. pylori* ATCC 43504 in the absence and presence of capsaicin. The data presented is the average of four replicates with SD. (**B**) Inhibition of *H. pylori* ATCC 43504 biofilm development by capsaicin. The data presented is the average of four replicates with SD. Secondary y-axis shows % inhibition with respect to control. Statistical significance was assessed using one-way analysis of variance (ANOVA) followed by Dunnett’s post hoc test for multiple comparisons with statistical significance defined as *p* < 0.05. ns is not significant and *** is *p* < 0.001. (**C**) Microscopic analysis of *H. pylori* ATCC 43504 biofilms in the absence and presence of capsaicin. (**C-i**) Light microscopic images. (**C-ii**) Confocal microscopic images with respect to control.

The inhibitory effect of capsaicin on the growth kinetics of *H. pylori* ATCC 43504 was quantified by calculating the percentage growth inhibition relative to the untreated control at each time point (Figure 1A). Control and solvent cultures exhibited typical growth curves, reaching OD_600_ values of 0.711 ± 0.023 and 0.720 ± 0.032, respectively at 48 h, confirming the bacterial growth and negligible solvent effect. Capsaicin treatment resulted in a clear concentration-dependent suppression of growth. At 6 h, inhibition was already evident at higher concentrations, with 400 µM showing 85.14% inhibition and 200 µM showing 78.75% compared to control. At 12 h, inhibition remained strong (92.21% at 400 µM, 87.32% at 200 µM), while moderate inhibition was observed at 150 µM (40.15%). By 24 h, 400 µM maintained 95.37% inhibition, 200 µM showed 89.02%, and 150 µM showed 32.57%, whereas 100 µM showed only 8.34% inhibition. At 48 h, 400 µM and 200 µM continued to suppress growth (92.45% and 90.85% inhibition, respectively), while 150 µM showed 24.87% inhibition. After 48 h and 100 µM, there was an insignificant inhibition of 3.67%. These findings demonstrate that capsaicin exerts potent growth inhibition against *H. pylori*, with 400 µM and 200 µM doses maintaining >90% inhibition throughout the growth cycle. Hence, the doses of 100 µM and below were selected for further biofilm studies as 100 µM capsaicin does not inhibit bacterial growth. This analysis confirms that capsaicin interferes with the growth of *H. pylori* in a dose-dependent manner. At lower concentrations, capsaicin delays bacterial growth, consistent with a bacteriostatic effect. At higher concentrations, near-complete suppression of bactericidal growth occurs. These findings align with earlier reports showing that capsaicin inhibited the growth of *H. pylori* in time- and dose-dependent manners [29].

### 3.2. Capsaicin Inhibits Biofilms of H. pylori

To evaluate the antibiofilm potential of capsaicin against *H. pylori* ATCC 43504, biofilm formation was examined using crystal violet assay. Capsaicin treatment resulted in a clear dose-dependent inhibition of biofilm biomass compared to untreated and solvent controls, as shown in Figure 1B. The highest concentration tested (100 µM) achieved 75.66 ± 4.00% inhibition, while lower concentrations (50 µM, 25 µM, and 10 µM) showed 52.67 ± 5.58%, 36.97 ± 7.45%, and 14.21 ± 4.92% inhibition, respectively. The solvent (0.5% DMSO) exhibited negligible inhibition, validating that the observed effects are due to capsaicin. These results demonstrate that capsaicin effectively suppresses biofilm formation in a concentration-dependent manner with remarkable inhibition. *H. pylori* is a persistent gastric pathogen strongly associated with peptic ulcers, gastritis, and gastric carcinoma [30]. One of the key survival strategies of *H. pylori* is its ability to form biofilms on gastric mucosa, which protect the bacteria from hostile gastric conditions, host immune responses, and antibiotic treatment [5]. Biofilm formation not only enhances colonization but also contributes to chronic infection and therapeutic failure [31]. Therefore, targeting biofilm development represents a promising approach to control *H. pylori* infections. Capsaicin’s inhibitory effect may be attributed to interference with quorum sensing pathways and reduction in cell surface hydrophobicity, thereby impairing bacterial adhesion and biofilm maturation.

### 3.3. Microscopic Analysis of Biofilm Inhibition

Microscopic visualization was performed to confirm the inhibitory effect of capsaicin on *H. pylori* ATCC 43504 biofilms grown on glass coverslips (Figure 1C). Light microscopy of untreated samples revealed dense bacterial colonization forming thick, continuous biofilm layers. In contrast, capsaicin-treated samples showed visibly reduced colonization, with thinner and disrupted biofilm layers. The biofilm was markedly diminished compared to controls. Confocal microscopy provided detailed insights into biofilm architecture. Untreated biofilms displayed compact bacterial aggregation embedded in an EPS. Capsaicin treatment led to significant disruption of biofilm architecture, with reduced thickness, scattered bacterial clusters, and diminished EPS matrix.

Microscopic observations corroborate the quantitative inhibition assays, demonstrating that capsaicin reduces biofilm biomass. The inhibition of biofilms suggest that capsaicin interferes with bacterial adhesion and EPS production, weakening the protective matrix. In *H. pylori*, biofilm formation is a critical survival mechanism against gastric acidity and antibiotic stress [32]. Therefore, capsaicin’s ability to compromise biofilm integrity highlights its therapeutic potential. The microscopic evidence thus provides strong support for the action of capsaicin.

### 3.4. Effect of Capsaicin on Biofilm Metabolic Activity

**Figure 2:**
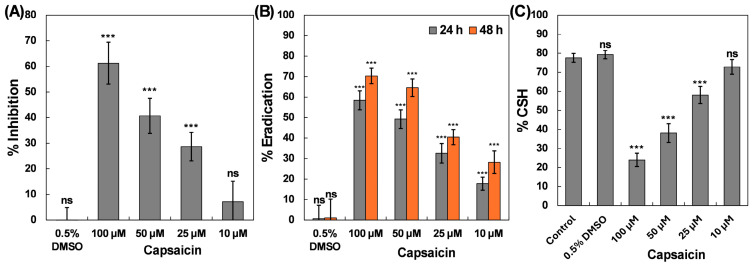
(**A**) Inhibition of biofilm metabolic activity in *H. pylori* ATCC 43504 by capsaicin. (**B**) Eradication of established *H. pylori* ATCC 43504 biofilms by capsaicin. (**C**) Cell surface hydrophobicity of *H. pylori* ATCC 43504 in the absence and presence of capsaicin. The data presented is the average of four replicates with SD. Statistical significance was assessed using one-way analysis of variance (ANOVA) followed by Dunnett’s post hoc test for multiple comparison with statistical significance defined as *p* < 0.05. ns is not significant and *** is *p* < 0.001 with respect to control.

The metabolic activity of *H. pylori* ATCC 43504 biofilms following capsaicin treatment was assessed using the XTT reduction assay. Capsaicin significantly reduced metabolic activity in the biofilm in a dose-dependent way compared to control (Figure 2A). At the lowest dose (10 µM), an insignificant inhibition of 7.14 ± 7.81% was found. The highest concentration (100 µM) achieved 61.23 ± 6.89% inhibition, while lower doses (50 µM and 25 µM) showed 40.69 ± 5.53% and 28.68 ± 8.02% inhibition, respectively. The solvent control (0.5% DMSO) exhibited negligible inhibition. These findings show that capsaicin reduces the metabolic activity of biofilm cells.

The reduction in metabolic activity suggests that capsaicin compromises the viability and physiological functions of *H. pylori* cells present in biofilm. Active metabolism is essential for *H. pylori* biofilms because it sustains the energy required for EPS production and maintenance of biofilm architecture [33]. Without active metabolic activity, biofilm cells lose their ability to adhere, communicate, and persist under hostile gastric conditions, leading to weakened biofilm integrity and reduced survival. The XTT assay highlights that capsaicin not only reduces biofilm formation but also impairs the metabolic activity in biofilms. This effect strengthens the evidence for capsaicin’s antibiofilm potential.

### 3.5. Eradication of Preformed Biofilm

The ability of capsaicin to eradicate pre-formed *H. pylori* ATCC 43504 biofilms was assessed at 24 h and 48 h post-treatment using the crystal violet assay. Capsaicin exhibited a clear time- and dose-dependent eradication effect (Figure 2B). At 24 h, the highest concentration (100 µM) achieved 58.43 ± 4.62% eradication, while 50 µM, 25 µM, and 10 µM showed 49.25 ± 4.54%, 32.62 ± 4.76%, and 17.77 ± 3.20%, respectively. At 48 h, eradication was further enhanced, with 100 µM reaching 70.31 ± 3.78%, 64.61 ± 4.28% at 50 µM, 40.48 ± 3.69% at 25 µM, and 28.23 ± 5.50% at 10 µM. The solvent control (0.5% DMSO) exhibited negligible activity (0.64 ± 6.57% at 24 h; 1.04 ± 9.10% at 48 h). These findings demonstrate that capsaicin is capable of both inhibiting biofilm formation and eradicating established biofilms. The eradication effect was evident at 24 h and further enhanced at 48 h post-treatment, indicating stronger activity upon prolonged exposure.

The eradication of mature biofilms is particularly challenging due to the protective extracellular matrix and altered physiology of biofilm-embedded cells [34]. *H. pylori* biofilm cells exhibit markedly reduced susceptibility to antibiotics compared to their planktonic growth. The biofilm-embedded cells were significantly less affected by clarithromycin and amoxicillin, highlighting the protective role of the biofilm matrix [35]. Capsaicin’s ability to significantly eradicate biofilm biomass at both 24 h and 48 h suggests that it penetrates the biofilm matrix and interferes with structural integrity. The enhanced eradication observed at 48 h indicates that prolonged exposure increases capsaicin’s efficacy, possibly by sustained disruption of cell–cell communication and weakening of matrix components. These findings reinforce the dual role of capsaicin in both preventing biofilm formation and dismantling established biofilms, making it a promising candidate for adjunctive against persistent *H. pylori* infections.

### 3.6. Hydrophobicity Index

The CSH of *H. pylori* ATCC 43504 was quantified using the microbial adhesion to hydrocarbons assay (Figure 2C). Untreated control cells exhibited high hydrophobicity (77.58 ± 2.31%). The 0.5% DMSO showed comparable SH of 79.27 ± 2.18%, confirming that the solvent had no significant effect. The lowest concentration of capsaicin (10 µM) produced a slight reduction (to 72.84 ± 3.85%) that was statistically insignificant compared to the control. In contrast, the highest concentration (100 µM) markedly decreased hydrophobicity to 23.96 ± 3.51%. These findings demonstrate that capsaicin significantly alters the physicochemical properties of *H. pylori* cells. The pronounced reduction in CSH at higher concentrations likely reflects alterations in outer membrane proteins and extracellular polymeric substances, consistent with impaired biofilm stability. Thus, capsaicin compromises the *H. pylori* CSH, which could limit biofilm formation and enhance susceptibility to antimicrobial agents.

### 3.7. Molecualr Docking of Capsaicin to Target Proteins

Molecular docking was performed to investigate the interactions of capsaicin with *H. pylori* GTP cyclohydrolase II (GCHII), α-carbonic anhydrase (α-CA), and urease. GCHII catalyzes the first step in riboflavin biosynthesis, and riboflavin derivatives (FMN/FAD) are essential cofactors for redox enzymes involved in energy metabolism and extracellular electron transfer. Riboflavin is known to induce anaerobic biofilm development in *Shewanella oneidensis* [36]. Carbonic anhydrases catalyze the reversible hydration of CO_2_, thereby maintaining pH homeostasis and bicarbonate balance in *H. pylori*. This activity supports acid tolerance and colonization, processes that indirectly stabilize biofilm formation [37]. Thus, while urease directly supports acid resistance, GCHII and α-CA represent metabolic and environmental adaptation pathways that may indirectly contribute to biofilm persistence in *H. pylori*.

**Figure 3:**
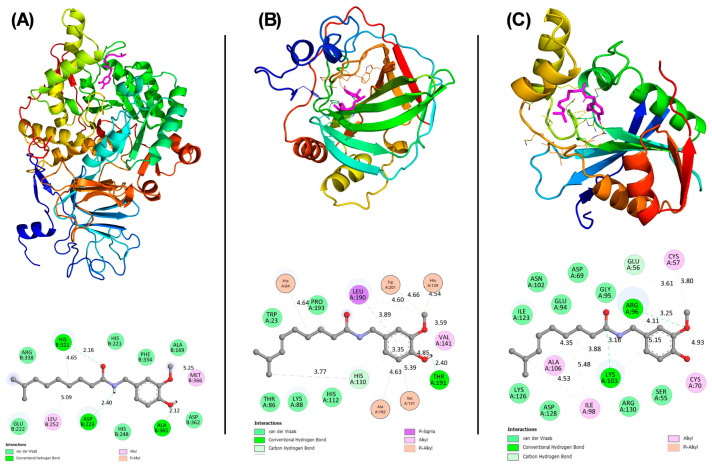
(**A**) Molecular docking of capsaicin with urease. In the top panel, urease is shown as rainbow ribbons and capsaicin is shown as magenta stocks. The bottom panel is a 2D interaction diagram. (**B**) Molecular docking of capsaicin with GTP cyclohydrolase II (GCHII). In the top panel, GCHII is shown as rainbow ribbons and capsaicin is shown as magenta stocks. The bottom panel is a 2D interaction diagram. (**C**) Molecular docking of capsaicin with α-carbonic anhydrase (α-CA). In the top panel, α-CA is shown as rainbow ribbons and capsaicin is shown as magenta stocks. The bottom panel is a 2D interaction diagram. The molecular docking was performed with AutoDock Vina.

To validate the docking protocol, the co-crystallized inhibitor (SHA) was extracted from the urease crystal structure and subsequently redocked. The ligand was docked at the same binding site as observed in the original crystal structure with a binding energy of −7.4 kcal/mol, thereby confirming the reliability of the docking approach. Molecular docking analysis revealed that capsaicin binds to *H. pylori* urease at the active site where its inhibitor SHA binds. The binding energy was found as −6.1 kcal/mol. The hydrogen bonds were observed with His322 (2.16 Å), Asp223 (2.40 Å), and Ala365 (2.12 Å), while van der Waals contacts involved Arg338, Phe334, Ala169, His221, Asp362, His248, and Glu222 (Figure 3A). Hydrophobic interactions with Leu252 (5.09 Å), Met366 (5.25 Å), and His322 (4.65 Å) further reinforced ligand stability within the active site pocket.

Molecular docking was performed to investigate the interactions of capsaicin with *H. pylori* GTP cyclohydrolase II (GCHII) and α-carbonic anhydrase (α-CA). Capsaicin was found to interact with both proteins, establishing multiple stabilizing interactions. The molecular docking of capsaicin with GCHII revealed a binding energy of −7.0 kcal/mol. For GCHI, capsaicin formed three hydrogen bonds with Lys103 (3.18 Å), Arg96 (3.25 Å), and Glu56 (3.61 Å) as shown in Figure 3B. The complex was further stabilized by van der Waals contacts with residues Lys126, Asp128, Arg130, Ser55, Gly95, Asp69, Glu94, Asn102, and Ile123. Hydrophobic interactions were observed with Ala106, Ile98, Cys70, and Cys57, suggesting strong non-polar contributions to binding. The binding energy for docking of capsaicin with α-CA was found to be −5.9 kcal/mol. For α-CA, capsaicin established hydrogen bonds with Thr191 (2.40 Å) and His110 (3.35 Å). Van der Waals interactions were noted with Thr86, Lys88, His112, Trp23, and Pro193 (Figure 3C). Hydrophobic contacts were formed with His84, Leu190, Trp201, His129, Val141, Val131, and Ala192. These interactions indicate that capsaicin fits well into the binding pocket of α-CA, stabilized by a combination of polar and non-polar forces.

The docking results demonstrate that capsaicin interacts with urease, GCHII, and α-CA through hydrogen bonds, van der Waals contacts, and hydrophobic interactions. In urease, strong hydrogen bonds with His322, Asp223, and Ala365, together with stabilizing hydrophobic contacts, suggest that capsaicin can effectively anchor within the catalytic pocket and potentially impair enzymatic activity essential for acid resistance. For GCHII, the presence of multiple hydrogen bonds indicates robust polar stabilization, while in α-CA, extensive hydrophobic contacts highlight the importance of non-polar contributions to ligand binding. Collectively, these interactions imply that capsaicin may interfere with the catalytic functions of these enzymes, thereby weakening *H. pylori* survival strategies and disrupting biofilm formation.

### 3.8. Molecular Dynamics Simulations

**Figure 4:**
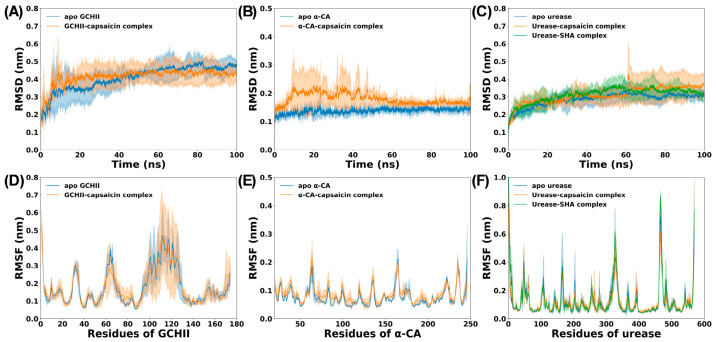
(**A**) Root mean square deviation (RMSD) of apo GCHII and GCHII–capsaicin complex over 100 ns MD simulation. (**B**) Root mean square deviation (RMSD) of apo α-CA and α-CA–capsaicin complex over 100 ns MD simulation. (**C**) Root mean square deviation (RMSD) of apo urease, urease–capsaicin complex, and urease–SHA complex over 100 ns MD simulation. (**D**) Root mean square fluctuation (RMSF) of GCHII in the absence and presence of capsaicin. (**E**) Root mean square fluctuation (RMSF) of α-CA in absence and presence of capsaicin. (**F**) Root mean square fluctuation (RMSF) of urease in the absence and presence of capsaicin or SHA. The data presented is the average of three simulations with thte4 shaded region being the SD. SHA is 2-{[1-(3,5-dimethylphenyl)-1H-imidazol-2-yl]sulfanyl}-N-hydroxyacetamide, which is a known inhibitor of urease.

Following the molecular docking studies, MD simulations were performed to assess the stability and conformational behaviour of target *H. pylori* proteins (GCHII and α-CA) in both apo and capsaicin-bound states. Root mean square deviation (RMSD) analysis was used to monitor structural deviations throughout the simulation trajectories. As shown in Figure 4A–C, all systems attained equilibrium within the initial few nanoseconds, indicating stable dynamics under simulated physiological conditions. The average RMSD values for apo GCHII and the GCHII–capsaicin complex were 0.406 ± 0.068 nm and 0.413 ± 0.044 nm, respectively, suggesting that capsaicin binding did not induce major structural perturbations. Similarly, apo α-CA exhibited a low RMSD of 0.137 ± 0.008 nm, which increased slightly to 0.180 ± 0.019 nm upon capsaicin binding, reflecting minor but acceptable deviations in backbone conformation. The apo urease showed an average RMSD of 0.285 ± 0.036 nm and the capsaicin- and SHA-bound complexes exhibited slightly higher but comparable deviations (0.310 ± 0.045 nm and 0.311 ± 0.040 nm, respectively).

Residue-level flexibility was examined using RMSF analysis of C_α_ atoms (Figure 4D–F). The RMSF profiles revealed that most residues in both proteins displayed fluctuations below 0.25 nm, consistent with stable protein dynamics. Apo GCHII residues fluctuated at 0.184 ± 0.109 nm, while the GCHII–capsaicin complex showed 0.186 ± 0.103 nm, indicating negligible influence of ligand binding. Apo α-CA residues fluctuated at 0.076 ± 0.032 nm, which increased marginally to 0.085 ± 0.030 nm in the complex. RMSF profiles of urease also revealed minimal fluctuations in residue mobility, with values remaining close between apo (0.120 ± 0.104 nm), capsaicin-bound (0.122 ± 0.095 nm), and SHA-bound (0.125 ± 0.126 nm) forms, suggesting that ligand binding did not induce major destabilization. Peaks in RMSF corresponded to loop and coil regions, which are inherently flexible in aqueous environments, and no significant destabilization was observed upon ligand binding [38].

**Figure 5:**
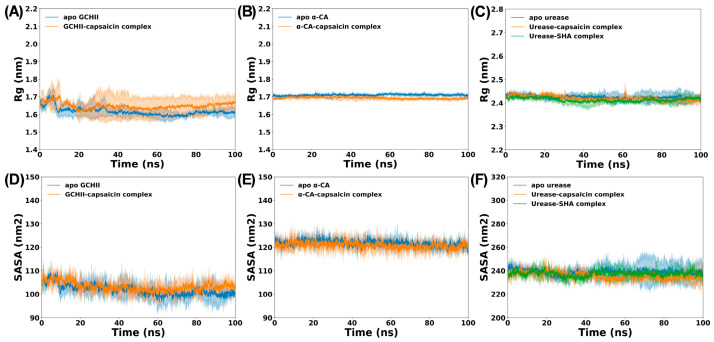
(**A**) Radius of gyration (Rg) of apo GCHII and GCHII–capsaicin complex over 100 ns MD simulation. (**B**) Radius of gyration (Rg) of apo α-CA and α-CA–capsaicin complex over 100 ns MD simulation. (**C**) Radius of gyration (Rg) of apo urease, urease–capsaicin complex, and urease–SHA complex over 100 ns MD simulation. (**D**) Solvent accessible surface area (SASA) of apo GCHII and GCHII–capsaicin complex over 100 ns MD simulation. (**E**) Solvent accessible surface area (SASA) of apo α-CA and α-CA–capsaicin complex over 100 ns MD simulation. (**F**) Solvent accessible surface area (SASA) of apo urease, urease–capsaicin complex, and urease–SHA complex over 100 ns MD simulation. The data presented is the average of three simulations with the shaded region being SD. SHA is 2-{[1-(3,5-dimethylphenyl)-1H-imidazol-2-yl]sulfanyl}-N-hydroxyacetamide, which is a known inhibitor of urease.

To evaluate structural compactness, the radius of gyration (R_g_) was calculated (Figure 5A–C). Apo GCHII recorded an average R_g_ of 1.616 ± 0.021 nm, which increased slightly to 1.647 ± 0.017 nm in the capsaicin complex, suggesting a minor expansion of protein structure. In contrast, apo α-CA exhibited an R_g_ of 1.709 ± 0.004 nm, which decreased nominally to 1.692 ± 0.005 nm upon capsaicin binding, indicating enhanced compactness. The R_g_ of urease also remained consistent across systems (2.425 ± 0.005 nm for apo, 2.421 ± 0.008 nm for capsaicin complex, and 2.413 ± 0.007 nm for SHA complex), confirming compact structural organization.

Solvent accessible surface area (SASA) analysis further supported these observations (Figure 5D–F). Apo GCHII displayed a SASA of 101.82 ± 2.19 nm^2^, which increased slightly to 103.19 ± 1.81 nm^2^ in the complex, while apo α-CA showed 121.67 ± 1.28 nm^2^, decreasing to 120.79 ± 1.21 nm^2^ upon ligand binding. Likewise, the SASA of urease showed a modest reduction upon ligand binding (238.781 ± 2.172 nm^2^ for apo urease, 235.840 ± 2.674 nm^2^ for urease–capsaicin complex, and 237.424 ± 2.103 nm^2^ for urease–SHA complex), indicating slight conformational adjustments that may reflect stabilization of the active site pocket. These subtle changes in SASA suggest minor alterations in solvent exposure, but overall structural integrity was maintained.

**Figure 6:**
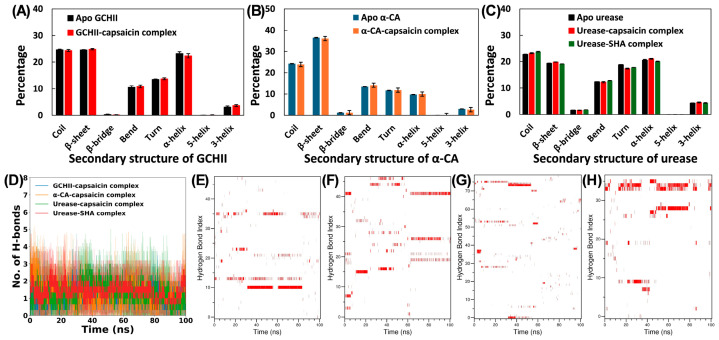
(**A**) Secondary structures in GCHII in the absence and presence of capsaicin. (**B**) Secondary structures in α-CA in the absence and presence of capsaicin. (**C**) Secondary structures in urease in the absence and presence of capsaicin or SHA. (**D**) Number of H-bonds formed by ligands (capsaicin or SHA) with GCHII, α-CA, or urease over 100 ns MD simulation. (**E**) Hydrogen bond existence map of GCHII–capsaicin complex over 100 ns MD simulation. (**F**) Hydrogen bond existence map of α-CA–capsaicin complex over 100 ns MD simulation. (**G**) Hydrogen bond existence map of urease–capsaicin complex over 100 ns MD simulation. (**H**) Hydrogen bond existence map of urease–SHA complex over 100 ns MD simulation. The data presented is the average of three simulations with the shaded region being SD. SHA is 2-{[1-(3,5-dimethylphenyl)-1H-imidazol-2-yl]sulfanyl}-N-hydroxyacetamide, which is a known inhibitor of urease.

To further evaluate the influence of capsaicin on the structural integrity of proteins, secondary structure analysis was performed following 100 ns MD simulations of both apo proteins and their capsaicin-bound complexes. The results, illustrated in Figure 6A–C, revealed that capsaicin binding did not induce any major perturbations in the overall secondary structure composition of either protein. In GCHII, the apo protein exhibited average percentages of coils (24.64 ± 0.20%), β-sheets (24.57 ± 0.10%), bends (10.54 ± 0.54%), turns (13.51 ± 0.14%), and α-helices (23.22 ± 0.67%). Upon complex formation with capsaicin, these values shifted only marginally to coils (24.35 ± 0.36%), β-sheets (24.84 ± 0.24%), bends (10.84 ± 0.52%), turns (13.74 ± 0.35%), and α-helices (22.35 ± 0.81%). The minor variations observed fall within the expected range of natural fluctuations, indicating that capsaicin binding does not substantially disturb the native folding pattern of GCHII. In the case of α-CA, the apo protein displayed secondary structure elements as coils (24.25 ± 0.06%), β-sheets (36.53 ± 0.09%), bends (13.42 ± 0.05%), turns (11.70 ± 0.06%), and α-helices (9.75 ± 0.04%). In the capsaicin-bound complex, these values remained nearly unchanged, with coils (23.90 ± 0.07%), β-sheets (36.13 ± 0.13), bends (14.15 ± 0.02%), turns (11.82 ± 0.05%), and α-helices (9.98 ± 0.05%). The negligible differences suggest that α-CA retains its native secondary structure organization even in the presence of capsaicin.

The apo urease displayed a distribution of structural elements, with coil (22.80 ± 0.06%), β-sheet (19.39 ± 0.05%), α-helix (20.54 ± 0.25%), and turn (18.87 ± 0.05%) as the major components. Upon capsaicin binding, a slight increase in coil (23.23 ± 0.17%) and α-helix (21.05 ± 0.16%) content was observed, accompanied by a reduction in turn structures (17.44 ± 0.15%). The SHA-bound complex showed similar trends, with increased coil (23.81 ± 0.14%) and bend (12.88 ± 0.05%) fractions, and a modest decrease in β-sheet (19.17 ± 0.04%) compared to the apo form. The MD simulations confirm that capsaicin binding does not cause significant alterations in the secondary structure of GCHII, α-CA, or urease. The proteins maintained their characteristic balance of coils, β-sheets, and helices, with only minor shifts in flexible regions such as bends and turns. The ability of capsaicin to bind stably while preserving native folding supports its potential to modulate enzymatic activity through localized interactions rather than global structural disruption, thereby contributing to its observed antibiofilm and growth inhibitory effects against *H. pylori*.

To better understand how capsaicin interacts at the molecular level with its target proteins, hydrogen bond dynamics were analyzed during the 100 ns MD simulations of the complex. The average number of hydrogen bonds formed between capsaicin and GCHII was 1.127 ± 0.401, while the α-CA–capsaicin complex exhibited a slightly higher average of 1.246 ± 0.565 (Figure 6D). The capsaicin-bound complex maintained an average of 1.28 ± 0.54 hydrogen bonds, while the SHA-bound complex exhibited a slightly higher average of 1.40 ± 0.43. These values indicate that both ligands form stable polar interactions with the active site residues, with SHA showing marginally stronger hydrogen bond stabilization. These values indicate that capsaicin maintained reasonably stable hydrogen bonding interactions with the target proteins throughout the simulation period. The hydrogen bond occupancy was also calculated (Figure 6E–H). In the GCHII–capsaicin complex, Asn102 exhibited maximum occupancy of 46.4%. Similarly, the highest H-bond occupancy of 27.0% was shown by Thr191 in the α-CA–capsaicin complex. Hydrogen bond occupancy analysis revealed that capsaicin maintained stable polar interactions within the urease active site, with Asp362 contributing 20.0% occupancy during the simulation. In comparison, the inhibitor SHA exhibited stronger stabilization at the same residue, with an occupancy of 39.7%. Although SHA demonstrated higher persistence, the ability of capsaicin to sustain consistent hydrogen bonding with a key catalytic residue shows its potential to interfere with urease function. Although the number of hydrogen bonds was modest compared to typical strong ligand–protein complexes, their persistence across the trajectory shows the ability of capsaicin to establish consistent polar contacts within the binding pockets. Moreover, the presence of stable hydrogen bonds complements the van der Waals and hydrophobic interactions identified in docking studies, collectively contributing to the overall stability of the complexes.

**Figure 7:**
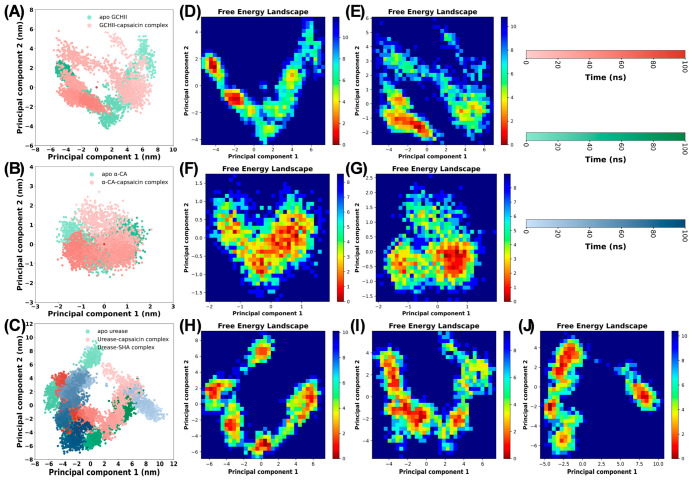
(**A**) Two-dimensional projection of eigenvectors of apo GCHII and the GCHII–capsaicin complex during 100 ns MD simulation. (**B**) Two-dimensional projection of eigenvectors of apo α-CA and the α-CA–capsaicin complex during 100 ns MD simulation. (**C**) Two-dimensional projection of eigenvectors of apo urease, the urease–capsaicin complex, and the urease–SHA complex during 100 ns MD simulation. (**D**) Free energy landscape of apo GCHII. (**E**) Free energy landscape of the GCHII–capsaicin complex. (**F**) Free energy landscape of apo α-CA. (**G**) Free energy landscape of the α-CA–capsaicin complex. (**H**) Free energy landscape of apo urease. (**I**) Free energy landscape of the urease–capsaicin complex. (**J**) Free energy landscape of the urease–SHA complex. SHA is 2-{[1-(3,5-dimethylphenyl)-1H-imidazol-2-yl]sulfanyl}-N-hydroxyacetamide, which is a known inhibitor of urease.

Principal component analysis (PCA) is a widely applied statistical approach for exploring large-scale motions and dynamic behaviour of proteins [39]. By reducing the dimensionality of simulation data, PCA identifies the dominant modes of motion, represented as eigenvectors, which capture the essential conformational variations in the system. In this study, PCA was employed to compare the flexibility of proteins in their apo state and in the complex with capsaicin. The two-dimensional projections of the first two eigenvectors are shown in Figure 7A–C. A broader distribution of conformational space in PCA plots reflects enhanced structural flexibility, whereas a more compact distribution indicates restricted motion. The analysis revealed that the capsaicin-bound complexes occupied conformational spaces comparable to those of the apo proteins, suggesting that ligand binding did not markedly alter the global flexibility of the systems. All target proteins (GCHII, α-CA, and urease) displayed similar eigenvector projections in the presence and absence of capsaicin, indicating that the complexes retained dynamic properties close to their native states.

To further investigate conformational stability, free energy landscapes (FELs) were constructed using the principal components (Figure 7D–J). The FELs demonstrated that all systems converged to well-defined energy minima, with only minor shifts in the positions of these minima upon ligand binding. The FEL analysis revealed distinct differences between apo and capsaicin-bound systems. A single energy minimum was observed in apo GCHII at 97.24 ns, and likewise in the GCHII–capsaicin complex at 94.12 ns, indicating that ligand binding did not substantially alter the conformational stability of this enzyme. In contrast, apo α-CA displayed one energy minimum at 96 ns, whereas the α-CA–capsaicin complex exhibited two separate minima at 38.4 ns and 48.64 ns, suggesting that capsaicin binding introduced additional conformational states and enhanced structural heterogeneity in α-CA. These findings highlight that while GCHII retains a stable energy landscape upon ligand binding, α-CA undergoes subtle conformational rearrangements in the presence of capsaicin. This observation supports the conclusion that capsaicin binding does not induce major destabilization, but rather maintains the overall energy landscape of the proteins.

To explore the thermodynamics of capsaicin binding, MM-PBSA analysis was carried out on the frames extracted from the last 60–100 ns of each molecular dynamics trajectory. The protein and ligand interaction is mainly established by non-covalent interactions such as van der Waals forces, electrostatic bonds, hydrophobic forces, and solvation energies, which together determine the binding affinity and stability of the complex [40]. The MM-PBSA energy profiles are summarized in Table 2. For the GCHII–capsaicin complex, van der Waals interactions emerged as a dominant stabilizing force, contributing −32.776 ± 4.295 kcal/mol. Electrostatic forces also provided favourable contributions (−9.032 ± 0.940 kcal/mol), while SASA energy contributed modestly (−4.143 ± 0.060 kcal/mol). In contrast, polar solvation energy consistently showed positive values (31.159 ± 2.840 kcal/mol), indicating an unfavourable contribution to binding. The overall binding free energy was calculated as −14.802 ± 3.682 kcal/mol, confirming that capsaicin forms a stable complex with GCHII. For the α-CA–capsaicin complex, a similar trend was observed. Van der Waals forces contributed −29.037 ± 3.938 kcal/mol, while electrostatic interactions were slightly stronger than in GCHII (−10.203 ± 3.140 kcal/mol). SASA energy again played a minor stabilizing role (−3.709 ± 0.171 kcal/mol), whereas polar solvation energy was unfavourable (26.721 ± 6.309 kcal/mol). The computed binding free energy was −16.231 ± 2.168 kcal/mol, suggesting that capsaicin binds α-CA with slightly higher affinity compared to GCHII. The complexes of urease exhibited comparable total binding free energies, with capsaicin showing −15.59 ± 1.72 kcal/mol and SHA as −15.87 ± 2.76 kcal/mol. The van der Waals contributions were the dominant stabilizing factor (−29.41 ± 3.42 kcal/mol for capsaicin and −28.43 ± 7.05 kcal/mol for SHA), while electrostatic interactions provided additional favourable contributions (−8.69 ± 5.29 kcal/mol and −9.91 ± 4.64 kcal/mol, respectively). Polar solvation energies were positive in both cases, indicating an unfavourable component, whereas SASA energies contributed modest stabilization (−3.87 ± 0.41 kcal/mol for capsaicin and −3.48 ± 0.64 kcal/mol for SHA).

In addition to global binding energy calculations, MM-PBSA was employed to estimate the individual residue-wise energy contributions in the capsaicin–protein complexes. The results, summarized in Table 3, highlight the specific amino acids that play a main role in stabilizing capsaicin with GCHII and α-CA. For the GCHII–capsaicin complex, Ile123 (−1.337 ± 0.060 kcal/mol) emerged as the most significant contributor to binding energy, followed by Phe125 (−0.812 ± 0.046 kcal/mol) and Ile98 (−0.734 ± 0.032 kcal/mol). Additional stabilizing contributions were observed from Le58 (−0.432 ± 0.020 kcal/mol), Ala106 (−0.346 ± 0.024 kcal/mol), Met122 (−0.247 ± 0.024 kcal/mol), Asp128 (−0.212 ± 0.061 kcal/mol), Ile117 (−0.209 ± 0.037 kcal/mol), Leu63 (−0.183 ± 0.010 kcal/mol), and Gly99 (−0.156 ± 0.015 kcal/mol). In the binding of capsaicin with α-CA, Leu190 (−1.469 ± 0.005 kcal/mol) was identified as the strongest contributor, followed by Val131 (−0.921 ± 0.003 kcal/mol) and Ala192 (−0.894 ± 0.003 kcal/mol). Other residues such as Leu139 (−0.634 ± 0.004 kcal/mol), Val141 (−0.554 ± 0.001 kcal/mol), Lys22 (−0.458 ± 0.001 kcal/mol), Arg229 (−0.339 ± 0.001 kcal/mol), Trp23 (−0.283 ± 0.003 kcal/mol), His84 (−0.280 ± 0.003 kcal/mol), and Pro194 (−0.269 ± 0.003 kcal/mol) also contributed to stabilizing the ligand within the binding pocket. For the urease–capsaicin complex, Trp181 (−0.565 ± 0.028 kcal/mol), Leu318 (−0.514 ± 0.016 kcal/mol), Thr251 (−0.431 ± 0.025 kcal/mol), and Ile137 (−0.323 ± 0.009 kcal/mol) emerged as the most significant stabilizing residues, supported by additional contributions from Ala185, Ile140, Pro142, Asp250, Gly280, and Glu254. In contrast, the urease–SHA complex showed stronger stabilization from Met317 (−1.029 ± 0.055 kcal/mol), Leu318 (−0.778 ± 0.041 kcal/mol), Met366 (−0.727 ± 0.037 kcal/mol), and Cys321 (−0.671 ± 0.040 kcal/mol), with further contributions from Glu222, Asp316, Glu276, Val320, Thr251, and Asp250.

## 4. Conclusions

The findings of this study demonstrate that capsaicin effectively inhibits biofilm formation and disrupts established biofilm in *H. pylori*. Capsaicin treatment reduced biofilm development, metabolic activity in biofilms, and the cell surface hydrophobicity. The microscopic analyses revealed a marked disruption of biofilm architecture. Computational analysis provided detailed insights into capsaicin’s stable interactions with target proteins in *H. pylori*. The study provides a foundation for further investigations into capsaicin’s role in the management of biofilm-associated *H. pylori* infections. Although this study establishes the antibiofilm potential of capsaicin against *H. pylori*, future investigations are needed to address certain limitations like the effect of capsaicin on stomach acidic pH and its synergy with antibiotics and other biofilm inhibitors.

## Figures and Tables

**Table 1:** Details of the target proteins.

S. No.	Protein	PDB ID	Size of Grid			Centre of Grid		
			x	y	z	x	y	z
1.	GTP cyclohydrolase II (GCHII)	4RL4	40	44	58	−3.43	20.122	−11.109
2.	α-carbonic anhydrase (α-CA)	4XFW	50	50	52	23.571	1.845	22.556
3.	Urease	6ZJA	66	70	84	222.89	238.802	175.589

**Table 2:** MM-PBSA binding energies for interaction of ligands (capsaicin or SHA) with GCHII, α CA, or urease.

Energy	GCHII–Capsaicin Complex	α-CA–Capsaicin Complex	Urease–Capsaicin Complex	Urease–SHA Complex
van der Waal energy	−32.776 ± 4.295	−29.037 ± 3.938	−29.412 ± 3.419	−28.433 ± 7.053
Electrostatic energy	−9.032 ± 0.940	−10.203 ± 3.140	−8.690 ± 5.291	−9.906 ± 4.640
Polar solvation energy	31.159 ± 2.840	26.721 ± 6.309	26.379 ± 9.202	25.926 ± 8.594
SASA energy	−4.143 ± 0.060	−3.709 ± 0.171	−3.874 ± 0.405	−3.476 ± 0.638
Binding energy	−14.802 ± 3.682	−16.231 ± 2.168	−15.592 ± 1.724	−15.875 ± 2.759

SHA is 2-{[1-(3,5-dimethylphenyl)-1H-imidazol-2-yl]sulfanyl}-N-hydroxyacetamide, which is a known inhibitor of urease.

**Table 3:** Total energies of highest energy contributing residues of GCHII, α CA, or urease interacting with ligands (capsaicin or SHA).

GCHII–Capsaicin Complex		α CA–Capsaicin Complex		Urease–Capsaicin Complex		Urease–SHA Complex	
Residues	Total Energy	Residues	Total Energy	Residues	Total Energy	Residues	Total Energy
Ile123	−1.337 ± 0.060	Leu190	−1.469 ± 0.005	Trp181	−0.565 ± 0.028	Met317	−1.029 ± 0.055
Phe125	−0.812 ± 0.046	Val131	−0.921 ± 0.003	Leu318	−0.514 ± 0.016	Leu318	−0.778 ± 0.041
Ile98	−0.734 ± 0.032	Ala192	−0.894 ± 0.003	Thr251	−0.431 ± 0.025	Met366	−0.727 ± 0.037
Leu58	−0.432 ± 0.020	Leu139	−0.634 ± 0.004	Ile137	−0.323 ± 0.009	Cys321	−0.671 ± 0.040
Ala106	−0.346 ± 0.024	Val141	−0.554 ± 0.001	Ala185	−0.319 ± 0.020	Glu222	−0.460 ± 0.052
Met122	−0.247 ± 0.024	Lys22	−0.458 ± 0.001	Ile140	−0.315 ± 0.030	Asp316	−0.449 ± 0.020
Asp128	−0.212 ± 0.061	Arg229	−0.339 ± 0.001	Pro142	−0.303 ± 0.022	Glu276	−0.396 ± 0.025
Ile117	−0.209 ± 0.037	Trp23	−0.283 ± 0.003	Asp250	−0.281 ± 0.011	Val320	−0.392 ± 0.040
Leu63	−0.183 ± 0.010	His84	−0.280 ± 0.003	Gly280	−0.257 ± 0.013	Thr251	−0.383 ± 0.013
Gly99	−0.156 ± 0.015	Pro194	−0.269 ± 0.003	Glu254	−0.248 ± 0.023	Asp250	−0.366 ± 0.011

SHA is 2-{[1-(3,5-dimethylphenyl)-1H-imidazol-2-yl]sulfanyl}-N-hydroxyacetamide, which is a known inhibitor of urease.

## Data Availability

The data is available from the corresponding author on request.
